# Potato Cyst Nematodes: Geographical Distribution, Phylogenetic Relationships and Integrated Pest Management Outcomes in Portugal

**DOI:** 10.3389/fpls.2020.606178

**Published:** 2020-12-17

**Authors:** Maria João Camacho, Eugénia de Andrade, Manuel Mota, Filomena Nobrega, Claudia Vicente, Leidy Rusinque, Maria Lurdes Inácio

**Affiliations:** ^1^INIAV – National Institute for Agriculture and Veterinary Research, Oeiras, Portugal; ^2^NemaLab, MED – Mediterranean Institute for Agriculture, Environment and Development, Institute for Advanced Studies and Research, Universidade de Évora, Évora, Portugal; ^3^NemaLab, Departamento de Biologia, Escola de Ciências e Tecnologia, MED – Mediterranean Institute for Agriculture, Environment and Development, Universidade de Évora, Évora, Portugal; ^4^GREEN-IT Bioresources for Sustainability, ITQB NOVA, Oeiras, Portugal

**Keywords:** *Globodera pallida*, *Globodera rostochiensis*, *Solanum tuberosum*, disease, Heteroderidae

## Abstract

The identification and phylogenetic relationships of potato cyst nematodes (PCN) were studied to assess the potential value of geographical distribution information for integrated pest management of potato production in Portugal. This research focused on PCN species, *Globodera pallida* and *Globodera rostochiensis*. From 2013 until 2019, 748 soil samples from the rhizosphere of different potato cultivars were surveyed in the Portuguese mainland to detect and identify both species and track their location. PCN are widespread invasive species throughout Portugal. In fact, during the survey period an incidence of 22.5% was estimated for the tested samples. The patterns of infestation vary among regions, increasing from south to north, where PCN were first detected. Currently, both species are present in all potato producing regions of the country, with a greater incidence of *G. pallida*. Phytosanitary control measures are influencing to the observed results. The use of potato cultivars resistant *to G. rostochiensis* led to a decrease of this species but had no influence on *G. pallida* detections, which continues its reproduction freely since there are no effective resistant cultivars for this species. The relationship between the presence, infestation rate, spread and geographical distribution of PCN is discussed in terms of behavioral responses of the potato cultivars and the implications for developing new integrated crop protection measures.

## Introduction

**FIGURE 1:**
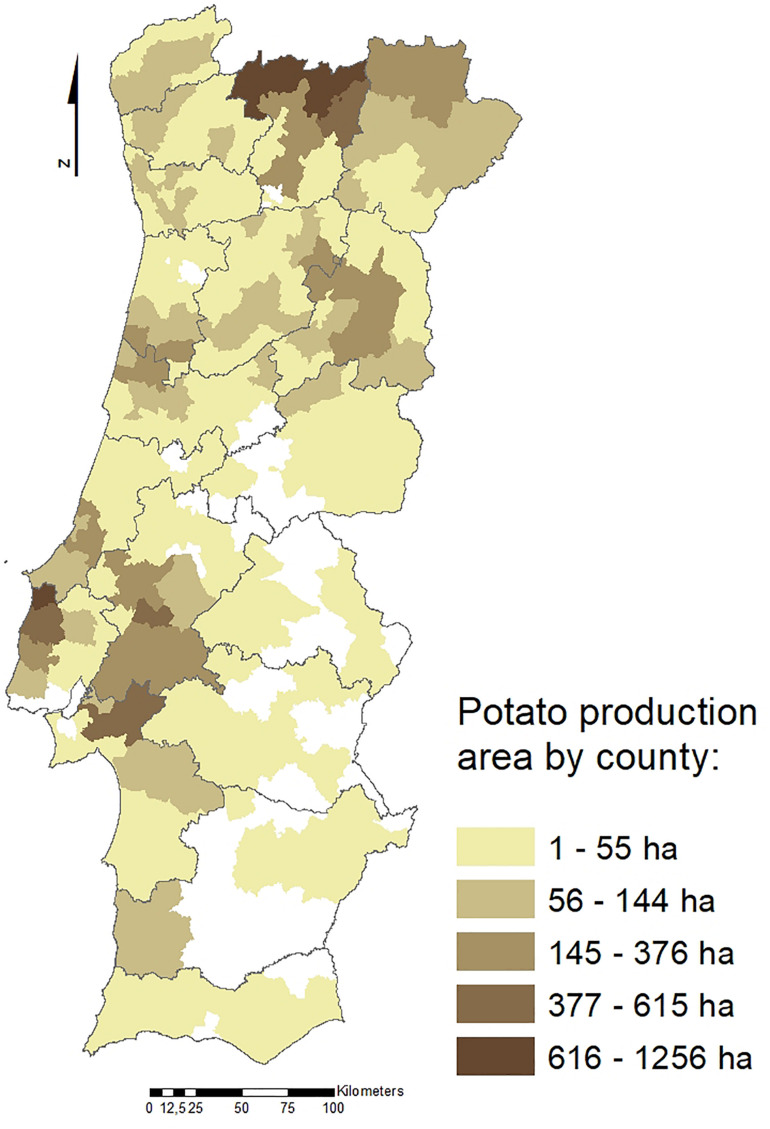
Potato production areas (ha) in Portugal by county (data from INE, 2011).

Potato crop (*Solanum tuberosum*) has great social and economic importance in Portugal since it is grown throughout the country. The most representative production regions are the North and West Regions (Figure 1), with a total potato growing area of approximately 20,000 hectares and a total production of 430,000 tons. Several nematode species have been reported associated with potato. Among those, the potato cyst nematodes (PCN), *Globodera rostochiensis* (Wollenweber, 1923; Skarbilovich, 1959) and *Globodera pallida* (Stone, 1973), are two of the major species limiting potato yield. These two species are sedentary endoparasites of the potato root system, deteriorate the quality and commercial value of tubers and contribute to infection of potatoes by other opportunistic plant pathogens, such as fungi (Lavrova et al., 2017).

Yield losses due to the presence of PCN, estimated at €220 million/year in Europe (Viaene, 2016), can vary from slight losses to crop failure depending on the infestation level (Lima et al., 2018).

Both PCN species are considered harmful quarantine organisms and are subject to stringent regulatory measures when detected singly or in combination (EPPO, 2017).

The golden potato cyst nematode, *G. rostochiensis*, and the pale potato cyst nematode, *G. pallida*, originated from the Andes region in southern Peru and have spread as the result of anthropogenic activity into many regions of the world (Grenier et al., 2010). They are thought to have been introduced to Europe in the 16–17th century by means of potato tubers carrying infested soil and nowadays have worldwide distribution. PCN have been reported throughout Europe, South America and parts of Asia, North America, Oceania and Africa where potatoes are grown (EPPO, 2020). However, new *Globodera* sp. detections continue to be reported (Hafez et al., 2007; Mburu et al., 2018; Niragire et al., 2019; Inácio et al., 2020).

In Portugal, *G. rostochiensis* was first reported in 1956 (Macara, 1963) in a field of seed potatoes near Bragança (Trás-os-Montes district, North of Portugal) and is currently present in all potato producing regions of the country (DGAV, 2015; Camacho et al., 2017), including the Madeira and Azores islands (DGAV, 2015; Inácio et al., 2020). *Globodera pallida* was first identified in 1988 (Santos and Fernandes, 1988), also in Trás-os-Montes, but its current national distribution has not yet been reported.

The knowledge on the geographical distribution, density and spatial dynamics of pest populations is indispensable in integrated pest management (IPM) systems, as it raises considerable interest among plant breeders and plant pathologists for the need to better understand the interaction between pest or pathogen and host and to estimate the risk of crop damage. Therefore, information of PCN distribution and potato cultivars used is essential to understand the *Globodera* spp. regional range of expansion since their first report. As human activity is the most probable means of spreading PCN, there is a specific interest in the evaluation of the implemented control measures and their consequences to adopt more effective management practices.

Controlling PCN is a difficult task due to their high level of adaptation to the environment, the prolonged viability of cysts in the absence of the host plant for more than 20 years, either quiescent or diapause in the form of encysted eggs (Christoforou et al., 2014), and the risk of appearance of aggressive pathotypes in the monoculture of nematode-tolerant potato cultivars. To assess the prevalence and distribution of PCN species across the territory, a country-wide survey was established in 2010, outlining a new framework for phytosanitary protection measures against these harmful organisms to avoid dispersion in national and European Community territories and to ensure potato production of a guaranteed quality for consumers. The main potato growing regions of Portugal have been surveyed for the presence of *G. rostochiensis* and *G. pallida* since 2013.

Before the national survey started, infestations were almost entirely due to *G. rostochiensis* (Santos and Fernandes, 1988; Santos et al., 1995; Martins et al., 1996; Conceição et al., 2003, Cunha et al., 2004, 2006, 2012). The few *G. pallida* populations found in Portugal may suggest that it was introduced after *G. rostochiensis* or there were only few introductions that were kept confined by their low natural mobility. Recently, the analysis of soils sampled in Portuguese potato fields revealed a spread of *G. pallida* (Camacho et al., 2017). In case of PCN positive detection, growers have to choose one of the following options as a phytosanitary measure: (a) culture with a PCN-resistant potato cultivar for a 3-year quarantine period, (b) culture with non-host species or (c) uncultivated land for a 6-year quarantine period. The use of resistant cultivars must be done carefully, in order to prevent the increase of *G. pallida* populations, which are more difficult to control as there are only a few available resistant cultivars.

Currently, in Portugal, there is a lack of detailed information on the geographical distribution of potato cyst nematodes, the correlation between their pattern, the potato cultivars and the near future implications for potato production. Therefore, this study aims to: (i) gather all PCN detections data in Portugal; (ii) carry out a molecular characterization of Portuguese *Globodera* isolates based on sequences of the ITS-rRNA region; (iii) study the phylogenetic relationships of Globodera spp. isolates from Portugal; and (iv) correlate cyst infestations with potato cultivars used.

The research reported herein includes PCN isolates collected from Portuguese potato fields for the national PCN surveys from 2013 to 2019, which made it possible to obtain an accurate assessment of the incidence and phylogenetic relationship of the two PCN species in the territory and their spread in different PCN-resistant cultivars fields.

## Materials and Methods

### Sampling

Soil was collected during the surveys between 2013 and 2019. Sampling was conducted by official inspectors of the National Plant Protection Organization (DGAV, Portugal). According to Annex II of DL 87/2010, sampling consists of a randomized collection of a soil volume with 1500 ml of soil/ha, harvested at least 100 subsamples/ha, preferably in a rectangular mesh, not less than 5 m wide and no more than 20 m long between sampling points, covering the entire field. Soil samples were stored in plastic bags and individually coded by the official services to ensure the anonymity of the samples during the analysis period. Potato field location at the county level and potato cultivars used in these fields were accessed only after analysis results.

The detection, identification and infestation rate of the PCN species were related to their sample location, given by DGAV, and species positive detection maps were made using the ArcMap 10.6 software (ESRI, United States), CAOP2017_PORTUGAL and CAOPP2017_DISTRITOS shapefiles (DGT, 2017).

### *Globodera* spp. Molecular Identification

Cysts were extracted from soil samples using the Fenwick’s can method (Fenwick, 1940), according to the EPPO PM7/40 (3) protocol, isolated and counted under a binocular microscope (Leica MZ6, Germany). Cysts (1 to 20 depending on the sample infestation) containing eggs and juveniles were used for DNA extraction by means of the DNeasy Blood & Tissue Kit (Qiagen, Valencia, CA, United States) following the manufacturer’s instructions. The internal transcribed spacer region (ITS) of the ribosomal DNA repeat unit was amplified by duplex PCR for species identification. PCR reactions were performed in a 25 μL final volume using the Promega GoTaq Flexi DNA Polymerase Kit (Promega, Madison, United States), containing 1 μL template DNA, 5 μL GoTaq Flexi PCR buffer (2x), 1.5 mM MgCl_2_, 0.20 mM each dNTPs, 1.25 U GoTaq Flexi DNA Polymerase (Promega, Madison, United States) and 0.4 μM of each primer in a Biometra TGradient thermocycler (Biometra, Gottingen, Germany). The set of primers was composed of the forward primer ITS5 (5′-GGA AGT AAA AGT CGT AAC AAG G-3′) and the reverse PITSr3 (5′-AGC GCA GAC ATG CCG CAA-3′) for *G. rostochiensis* and PITSp4 (5′-ACA ACA GCA ATC GTC GAG-3′) for *G. pallida* (Bulman and Marshall, 1997). The amplification profile for ITS-rDNA consisted of an initial denaturation of 94°C for 2 min followed by 35 cycles of 94°C for 30 s, 55°C for 30 s, and 72°C for 30 s and a final extension of 72°C for 7 min (EPPO, 2017). The amplified products were loaded onto a 1.5% agarose gel containing 0.5 μg.mL^–1^ ethidium bromide and 0.5× Tris-borate-EDTA (TBE) running buffer and electrophoresed at 5 V/cm. Amplifications were visualized using the VersaDoc Gel Imaging System (Bio-Rad, United States). The expected length of the PCR products was 265 bp for *G. pallida* and 434 bp for *G. rostochiensis*. Possible contaminations were checked by including negative controls (no template control – NTC) in all amplifications.

### *Globodera* spp. Phylogenetic Analysis

The ITS-rDNA region of 36 samples was amplified and sequenced using the primers 5′-CGT AAC AAG GTA GCT GTA G-3′ and 5′-TCC TCC GCT AAA TGA TAT G-3′ (Ferris et al., 1993). The expected length of PCR fragments is 1040 bp and corresponds to the 3′ end of 18S rDNA-ITS1-5.8S-ITS2-5′ of 28S rDNA. The thermal cycling conditions performed consisted of an initial denaturation of 95°C for 5 min followed by 40 cycles of 94°C for 30 s, 55°C for 30 s, and 72°C for 33 s and a final extension of 72°C for 7 min. Nucleotide sequences were edited and analyzed using BioEdit v7.2.0 (Hall, 2007). The resulting ITS-rDNA sequences were used as query at BLAST from NCBI GenBank to retrieve the most similar sequences within *Globodera* species for phylogenetic reconstruction, and they were deposited in the GenBank database (NCBI). Sequences from *Globodera artemisiae*, *Globodera tabacum*, and *Globodera hypolysi* were selected as outgroup taxa. All sequences were aligned by CLUSTAW (Thompson et al., 1994) with default parameters, trimmed manually and evaluated by Maximum Likelihood phylogeny using the best AIC (Akaike Information Criteria) nucleotide substitution model determined, namely Hasegawa-Kishino-Yano with Gamma Distribution (HKY + G). A bootstrap analysis with 1000 replications was also conducted to infer robustness of the phylogenetic tree. The CLC Main Workbench software package 8.1^1^ was used for phylogenetic analysis.

### Statistical Analysis

The differences obtained in the detection of the two PCN species in Portugal were achieved through a *Z*-test for the equality of two proportions using the software R^2^. Only soil samples with one or more cysts were used. The hypothesis tests were performed with a significance level α = 0.05.

Subsequently, the same test was used, with the same level of significance, to infer differences between PCN detections in north, center and south producing regions and between *G. pallida* and *G. rostochiensis* detections in fields with PCN susceptible and *G. rostochiensis* resistant potato cultivars.

## Results and Discussion

During the survey period (2013–2019), 748 soil samples were collected throughout the country by the official services and tested in the plant health national reference laboratory (INIAV). Potato cyst nematodes were identified in 168 samples, representing 22.5% of the tested samples. Forty-eight samples tested positive for *G. rostochiensis* populations alone (28.6%) and 83 for *G. pallida* populations alone (49.4%). Mixed populations were found in 37 samples (22%) (Table 1). Statistics revealed that two species detections are significantly different (*p*-value = 0.00014, α = 0.05), *G. pallida* detection being greater than *G. rostochiensis* detection (*p*-value = 0.999, α = 0.05, which allows us to accept the null hypothesis that *G. pallida* detections are significantly greater to *G. rostochiensis* detections) between 2013 and 2019. These results contrast with those reported by Cunha et al. (2004) in which out of 423 tested populations (samples collected from various districts of continental Portugal), 83% were *G. rostochiensis* populations alone, 8% were *G. pallida* populations alone and 9% consisted of a mixture of the two species. This reverse situation can be explained due to the use of *G. rostochiensis* resistant potato cultivars, which has been considered the most widespread PCN species in Portugal.

**TABLE 1:** Samples tested for *Globodera rostochiensis* and *Globodera pallida* in Portuguese regions between 2013 and 2019 (absolute values and %).

**Region**	**Positive detections**								**Negative detections**		**Total**
	***G. rostochiensis***		***G. pallida***		***Gr* + *Gp***		**Total**				
						
	**Value**	**%**	**Value**	**%**	**Value**	**%**	**Value**	**%**	**Value**	**%**	
North	30	40.5	39	52.7	5	06.8	74	42.5	100	57.5	174
Center	11	18.0	32	52.5	18	29.5	61	25.5	178	74.5	239
South	7	21.2	12	36.4	14	42.4	33	9.9	302	90.1	335
Total	48	28.6	83	49.4	37	22.0	168	22.5	580	77.5	748

The use of *G. rostochiensis* resistant potato cultivars (Table 2), effective only against certain races of *G. rostochiensis* and with no resistance to *G. pallida*, is leading to the predominance in Portugal of the more difficult species to control, *G. pallida*. The obtained *p*-value (*p*-value = 0.996, α = 0.05) supported the null hypothesis, confirming that *G. rostochiensis* detection in potato fields with *G. rostochiensis* resistant cultivars is significantly smaller than *G. rostochiensis* detection in potato fields with PCN susceptible cultivars. With this result it is possible to infer that resistant cultivars are more efficient in reducing cyst infestations in potato production fields compared with susceptible cultivars fields. However, *G. pallida* detection in potato fields with *G. rostochiensis* resistant cultivars is not different to *G. pallida* detection (*p*-value = 0.2048, α = 0.05, which allows us to accept the null hypothesis that *G. pallida* detections in *G. rostochiensis* resistant cultivars are significantly similar to *G. pallida* detections in PCN susceptible cultivars) and *G. rostochiensis* detection in PCN susceptible potato cultivars fields (*p*-value = 0.5415, α = 0.05, which allows us to accept the null hypothesis that *G. pallida* detections in *G. rostochiensis* resistant cultivars are significantly similar to *G. rostochiensis* detections in PCN susceptible cultivars). With this result it is possible to infer that resistant cultivars used in Portugal allow us to reduce *G. rostochiensis* cysts infestation but has no influence on *G. pallida* cysts infestations in potato production fields. Therefore, the use of *G. rostochiensis* resistant potato cultivars has led to a decrease in *G. rostochiensis* detection but has no influence on *G. pallida* detection. These results agree with the published literature (Minnis et al., 2002; Pickup et al., 2019).

**TABLE 2:** Potato cultivars grown in Portuguese sampled fields (2013–2019) and their resistance status toward *Globodera rostochiensis* and *Globodera pallida.*

**Cultivar**	**Resistance status**			**Resistance status**	
	***G. rostochiensis***	***G. pallida***	**Cultivar**	***G. rostochiensis***	***G. pallida***
Agria	R	S	Jelly	R	S
Alcander	R	R	Kenebeck	S	S
Allison	R	R	Lady rosetta	R	S
Asterix	R	S	Manitou	R	S
Aurea	R	S	Monalisa	S	S
Baraka	R	S	Monte carlo	R	R
Bellarosa	R		Olho de perdiz	R	
Camberra	R		Picasso	R	
Carlita	R		Red Lady	R	
Colomba	R	S	Red scarlet	R	
Daifla	R	S	Romano	S	S
Delila	S	S	Rudolph	S	S
Désirée	S	S	Soleny	S	S
Evolution	R	S	Stemster	R	
Evora	S	S	Taurus	R	S
Hermes	S	S	Yona	R	S

*R, resistant; S, susceptible.*

There is no available data to infer about the use of *G. pallida* resistant potato cultivars. This raises the question of whether phytosanitary measures are effective or whether they are contributing to the increase of *G. pallida*, as also reported in the United Kingdom (Minnis et al., 2002). On the other hand, the market has caused potato growers to predominantly use *G. rostochiensis* resistant potato cultivars (i.e., Aurea, Agria, Lady rosetta, Taurus), and this is the main cause of *G. pallida* detections increase.

**FIGURE 2:**
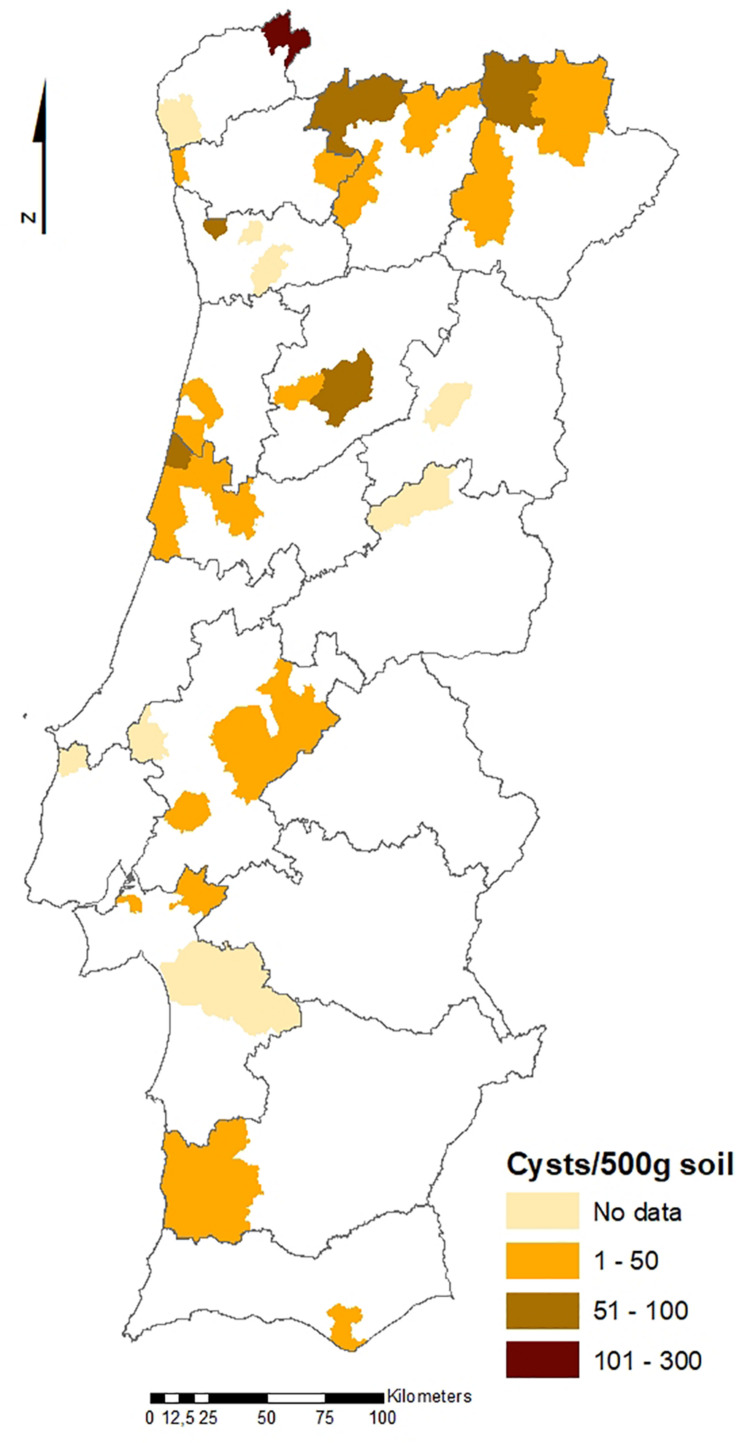
Counties with positive detections of *Globodera rostochiensis* in Portugal between 2013 and 2019.

**FIGURE 3:**
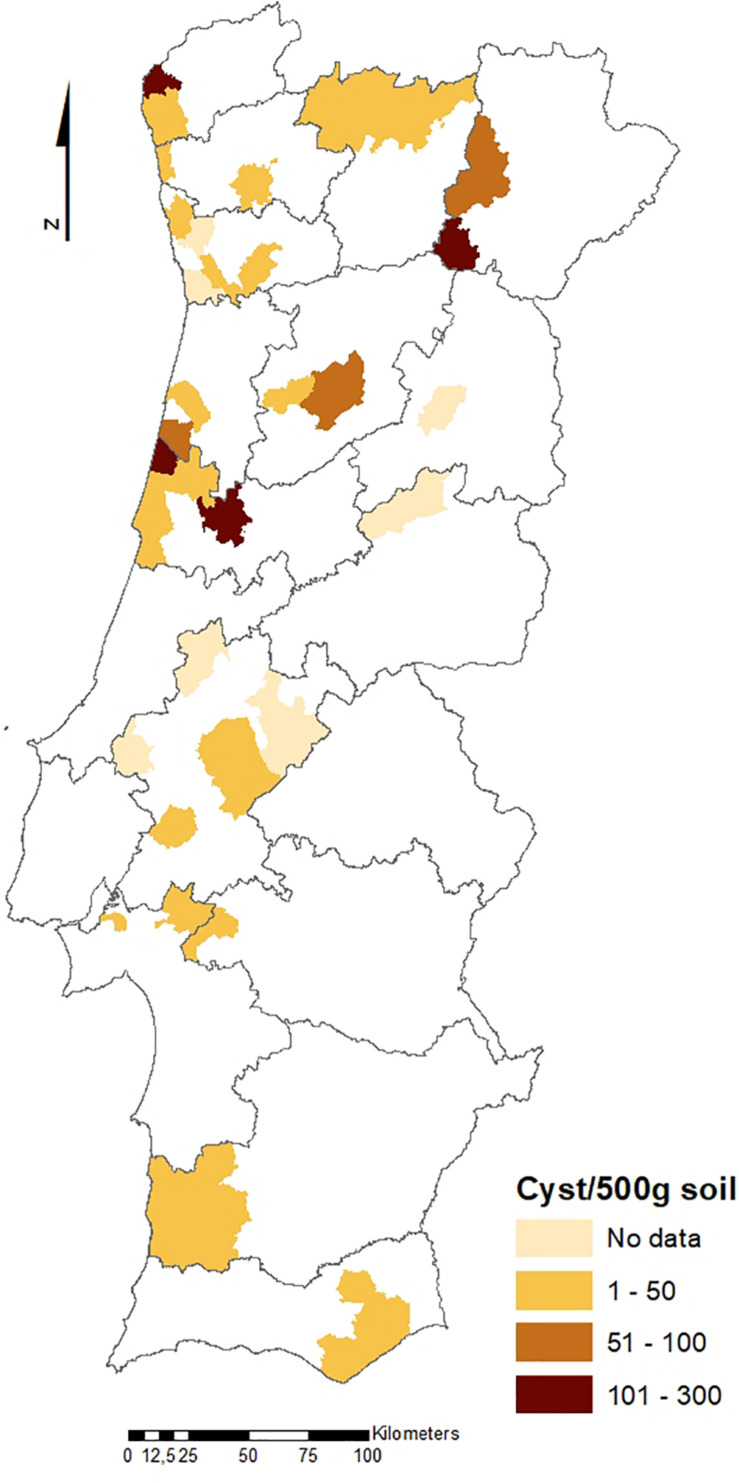
Counties with positive detections of *Globodera pallida* in Portugal between 2013 and 2019.

The geographical distribution of PCN infestations in Portugal is illustrated in Figures 2, 3, which present the infestation rate in counties with positive detections of *G. rostochiensis* and *G. pallida* between 2013 and 2019. This information completes a picture of the PCN situation in Portugal to date.

According to these results, the incidence of PCN in Portugal is quite high, and both species are currently present in all potato producing regions of the country. PCN detections in the different regions are significantly different. Statistics revealed that the Northern PCN detection is greater than the Center PCN detection (*p*-value = 0.998, α = 0.05, which allows us to accept the null hypothesis that PCN detection in northern fields is significantly greater than PCN detection in central fields) and the Center PCN detection is greater than the Southern (Lisbon and Tagus Valley, Alentejo and Algarve regions) PCN detection (*p*-value = 1, α = 0.05, which allows us to accept the null hypothesis that PCN detection in central fields is significantly greater than the PCN detection in southern fields), meaning that PCN detection increases from south to north (see Figures 2, 3), where PCN were first detected and nematode reproduction are happening for a longer period. These results are also in line with previous reports, which state that the cysts are adapted to higher altitudes (Jones et al., 2017) since the altitude grows from south to northern regions in Portugal.

**FIGURE 4:**
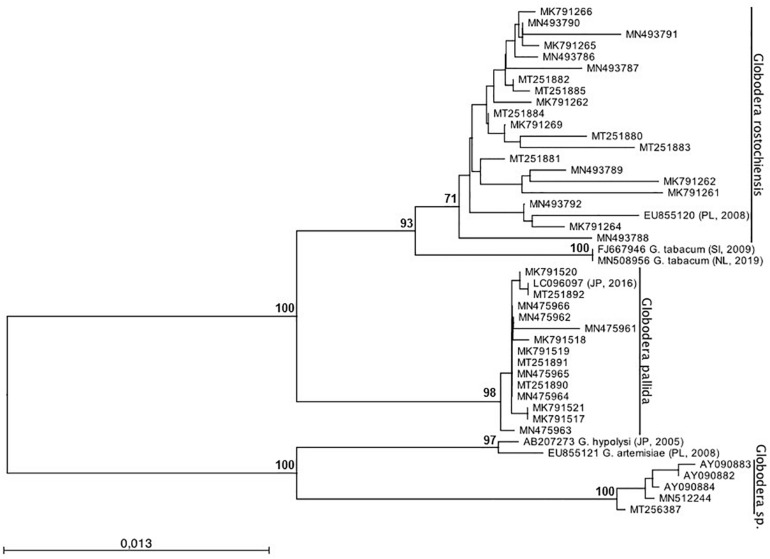
Phylogenetic relationships of 36 *Globodera* sp. isolates collected from Portugal based on the sequence alignment of the ITS-rDNA loci. The condensed phylogenetic tree was generated using the Maximum Likelihood method based on the HKY + G model with 1,000 bootstrap replications. Bootstrap values are indicated at the nodes (bootstrap higher than 70%). The analysis involved a total of 45 nucleotide sequences. All positions containing gaps and missing data were eliminated. *Globodera tabacum*, *G. hypolysi*, and *G. artemisiae* were used as outgroup.

To infer the phylogenetic relationship of *Globodera* isolates, ML analyses were performed (Figure 4). Two major clades, highly supported, can be observed: clade (I) with sub-clades *G. rostochiensis* and *G. pallida* and clade (II) with the sub-clades *Globodera* sp. recently re-detected. Within the first clade, two sub-clades were formed with *G. rostochiensis* and the related species *G. tabacum* and *G. pallida*. The second clade groups a Portuguese *Globodera* sp., discovered in 1997 (Reis, 1997; Sabo et al., 2002) and not re-detected until recently (data not shown), and their most closely related *Globodera* species, *G. hypolysi* and *G. artemisiae*. As can be clearly seen, no spatial-temporal relation can be redrawn evidencing the co-existence between the two major species of *Globodera* in Portugal. These results are in accordance with those reported by Cunha et al. (2012), who reported that no relationship could be found between the two-dimensional electrophoresis protein patterns or virulence behavior of the isolates and their geographic origin within Portugal.

It is also worth noting that the topology differs between *G. rostochiensis* and *G. pallida* sub-clades. The first is more branched, with 96–100% of similarity, showing more genetic variability due to being present for a longer period in Portugal, while the second is flatter, with 99–100% of similarity, showing more identical sequences (Supplementary Table 1).

Concerning the new species *Globodera* sp. (Reis, 1997; Sabo et al., 2002), re-detected recently in Portugal, it is out of the scope of this work, but additional research is being carried out to determine its pathogenicity and impact on potato.

The nucleotide sequences obtained in this study were deposited in the GenBank database (NCBI) under the accession numbers given in Table 3.

**TABLE 3:** *Globodera* spp. isolates sequenced in the present study (*E*-value = 0.0).

***Globodera* species**	**GenBank accession number**	**Locality**	**Collection code/year**		**Sequence length (bp)**	**NCBI BLAST homology (%)**
*Globodera rostochiensis*	EU855120	Poland	*	2008	4064	100.00
	MK791260	Coimbra	650P	2014	893	100.00
	MK791261	Montalegre	5244	2015	888	100.00
	MK791262	Montalegre	5245	2015	909	100.00
	MK791263	Viseu	9996	2018	871	98.62
	MK791264	Mirandela	14598	2018	969	99.79
	MK791265	Mirandela	14600	2018	871	99.89
	MK791266	Bragança	14601	2018	909	99.89
	MN493786	Montalegre	13486	2017	937	99.25
	MN493787	Chaves	8850	2016	937	98.50
	MN493788	Viseu	9610	2017	920	98.58
	MN493789	Viseu	5967	2016	936	98.82
	MN493790	Viseu	7047	2017	973	100.00
	MN493791	Odemira	3663	2018	915	99.13
	MN493792	Aveiro	7913	2018	897	99.78
	MT251880	Coimbra	1252	2019	929	99.14
	MT251881	Montalegre	1681-2	2019	909	99.34
	MT251882	Montalegre	1681-6	2019	924	99.89
	MT251883	Chaves	1681-7	2019	933	98.71
	MT251884	Mirandela	1681-10	2019	928	99.35
	MT251885	Melgaço	1249-1	2019	946	98.94
*Globodera tabacum*	FJ667946	Slovenia	*	2009	923	99.46
	MN508956	Netherlands	NL:c6876	2018	953	99.89
*Globodera pallida*	LC096097	Japan	*	2016	964	100.00
	MN475961	Viseu	3876	2014	898	99.33
	MN475962	S. Magos	4261	2016	970	99.90
	MN475963	S. Magos	15731	2018	933	99.03
	MN475964	Vagos	9993	2018	977	98.89
	MN475965	Montalegre	14002	2017	914	99.89
	MN475966	Esposende	5087	2016	926	99.56
	MK791517	Penafiel	4694	2015	873	100.00
	MK791518	Viseu	5961	2016	890	99.22
	MK791519	Guimarães	11309	2018	901	99.78
	MK791520	Mirandela	14593	2018	878	100.00
	MK791521	Mirandela	14599	2018	873	100.00
	MT251890	Vagos	1223-7	2019	938	100.00
	MT251891	Aveiro	1223-8	2019	915	99.89
	MT251892	Mira	1086-3	2019	913	99.67
*Globodera* sp.	AY090883	Bouro	*	1997	908	99.89
	AY090882	Canha	*	1997	908	99.89
	AY090884	Ladoeiro	*	1997	908	99.78
	MN512244	Montijo	12031	2018	953	99.45
	MT256387	Lagameças	1479-2	2019	913	99.67
*Globodera artemisiae*	EU855121	Poland	*	2008	4092	100.00
*Globodera hypolysi*	AB207273	Japan	*	2005	909	99.45

*Sequences available from GenBank, NCBI.

Phytosanitary measures have been taken to prevent further spread of *Globodera* spp. in recent years. In the case of *G. rostochiensis*, up until now the dominant species, measures include non-host crop rotation (for 6 years), fallow (for 6 years) or growing of resistant potato cultivars (for 3 years). The use of resistant cultivars containing the H1 gene (single dominant resistance gene for *G. rostochiensis*) (Gebhardt et al., 1993), as already shown, is effective against many populations of *G. rostochiensis* and is likely to be an advantageous management tactic to reduce population densities and thereby yield losses. However, the deployment of resistance in such cultivars may have caused the predominance of *G. pallida* in Portugal, as already predicted by Cunha et al. (2004) and statistically verified in this study.

Therefore, it is urgent to follow a new approach for the management of PCN, mainly *G. pallida*. Non-infested areas need to be managed to minimize the opportunities for the introduction of *Globodera* species. On the other hand, and in infested soils, a greater use of integrated control strategies (such as crop rotation, solarization, trap cropping, biofumigation and selected nematicides) (Evans and Haydock, 2000; Alptekin, 2011; Davie et al., 2019), in addition to PCN-resistant potato cultivars, should be a priority. These interactions require careful research into the effects of one or another strategy under a specific set of environmental conditions and a specific nematode infestation level. The efficacy of the integrated program will be determined by the interaction, overlap and complementarity of the various components. Despite the difficulties associated with *G. pallida* resistance being quantitatively inherited, the breeding of more resistance with different R-genes to avoid PCN capacity to overcome the plant resistance and commercially attractive cultivars is highly important. As *G. pallida* field populations tend to show increased virulence toward a particular partially resistant cultivar each time that it is grown (Trudgill et al., 2003; Pickup et al., 2019), potato growers would need a choice of different cultivars to allow effectiveness to be maintained. Currently, there are insufficient alternatives to partially resistant cultivars for growers to meet the requirements of markets.

## Data Availability Statement

The datasets presented in this study can be found in online repositories. The names of the repository/repositories and accession number(s) can be found in the article/Supplementary Material.

## Author Contributions

MC, MI, EA, and MM: conceptualization. MC: investigation and writing – original draft. MC, MI, EA, FN, CV, and LR: methodology. MI, EA, and MM: supervision. MC, MI, EA, MM, and CV: writing – review and editing. All authors contributed to the article and approved the submitted version.

## Conflict of Interest

The authors declare that the research was conducted in the absence of any commercial or financial relationships that could be construed as a potential conflict of interest.

## Footnotes

1 https://www.qiagenbioinformatics.com/

2 https://www.r-project.org/

## Supplementary Material

Supplementary Table 1 Sequence percentage of similarity.
